# Physicians’ Perceptions of, and Satisfaction with, Electronic Medical Record Systems in St. Paul’s Hospital Millennium Medical College, Addis Ababa, Ethiopia

**DOI:** 10.3390/healthcare14152379

**Published:** 2026-08-04

**Authors:** Simon Fitehamlak Yihune, Dereje Bayissa Demissie, Hanan Ali

**Affiliations:** 1 School of Medicine, St. Paul’s Hospital Millennium Medical College, Addis Ababa P.O. Box 1271, Ethiopia; simfthamlk37@gmail.com (S.F.Y.); hanan.ali@sphmmc.edu.et (H.A.); 2 Department of Medical Neonatal, School of Nursing, St. Paul’s Hospital Millennium Medical College, Addis Ababa P.O. Box 1271, Ethiopia

**Keywords:** electronic medical records, physicians’ perceptions, physicians’ satisfaction

## Abstract

**Background:** This study explores physicians’ perceptions and satisfaction with Electronic Medical Record (EMR) systems in resource-limited healthcare settings, addressing a critical gap in research. It identifies key factors essential for optimizing EMR adoption and enhancing healthcare delivery. **Methods:** This study employed a facility-based mixed-method cross-sectional study comprising a quantitative survey of 249 physicians at St. Paul’s Hospital Millennium Medical College (SPHMMC), sampled through stratified random sampling techniques, with thematic analysis of semi-structured interviews of eight physicians, selected using criterion and stratified purposive sampling techniques. Quantitative data was analyzed using both binary and multivariable logistic regression, and qualitative data from semi-structured interviews was analyzed using thematic analysis. **Results:** The study reveals that 91% of physicians experience workflow disruptions due to EMR system use. Despite this, physicians were satisfied with efficiency gains associated with EMR systems, with 65.8% noticing improvements in their efficiency and 50.6% reporting reduced workloads. 78.7% of physicians expressed satisfaction with workflow integration. EMR communication tools were rated positively, with 92.0% satisfied with integration. Overall, 79.5% of St. Paul’s Hospital Millennium Medical College (SPHMMC) physicians are satisfied with the implemented EMR system, with factors such as ease of use, integration, confidence in navigation, sufficient technical support, and perceived effectiveness in facilitating patient care across departments affecting satisfaction. The qualitative study also highlighted the following themes: a disconnect between system functionality and resource availability, usability and functionality gaps affecting workflow, inadequate training and support, data security and privacy concerns, and mixed perceptions of the EMR system’s impact on patient care benefits and challenges. **Conclusions:** The study shows that while the EMR system at SPHMMC has a mixed perception among physicians, it has generally satisfied them, and this supports a multifaceted approach to enhance physician satisfaction and optimize EMR utilization at SPHMMC. This includes enhancing system usability, providing comprehensive training, ensuring readily available technical support, improving workflow integration, and strengthening data security measures.

## 1. Background

Electronic Medical Record (EMR) systems are computerized systems employed in single healthcare facilities or medical practices to keep patient information pertaining to clinical visits. Such records commonly contain patient demographics, medication and prescription information, laboratory test results, and clinical notes made by healthcare practitioners in the process of delivering care [1].

A health record offers a lifelong, comprehensive view of an individual’s health, including lifestyle and personal data, while a medical record captures time-specific clinical care details [2].

Ancient Egyptian hieroglyphic inscriptions and papyri reveal evidence of medical recordkeeping. However, the use of paper medical records began between 1900–1920, and with computer technology advancements in the 1960s and 1970s, EMRs started to emerge [3]. The increased utilization of EMRs in the early 2000s was driven by their potential to improve healthcare by organizing patient data, enhancing care coordination, and simplifying access to medical information [4]. The increase in adoption of EMRs s over the last two decades is projected to persist, leading to the widespread use of EMR systems in hospitals within the next 10 to 15 years [5].

Developed countries, particularly in Europe and the United States of America (USA) have extensively adopted EMR systems to manage patient records and facilitate healthcare delivery. The widespread use of EMRs in these regions has led to centralized databases where different services and clinicians can quickly access patient data, thereby supporting healthcare delivery and improving clinical productivity, patient safety, and care quality [6,7,8].

In contrast, the adoption and usage of EMRs in developing countries are less common and, in many cases, non-existent. Healthcare providers in these regions often rely on paper-based records, which are inefficient and difficult to manage. The lack of a robust healthcare infrastructure, including information and communications technology, poses significant challenges to the implementation of EMR systems [6,7,9,10].

Currently, the Ethiopian Ministry of Health is trying to implement EMR systems that are aligned with the Ethiopian EMR standard, such as the Bahmni EMR system, in various governmental facilities, including at the University of Gondar Comprehensive and Specialized Hospital [11]. SPHMMC also utilizes the Bahmni EMR system, an open-source platform, to provide high-quality, patient-centered care in a resource-limited setting. Bahmni is a comprehensive hospital information system and EMR, designed for low-resource hospitals, offering integrated features like laboratory-, billing-, pharmacy-, and Picture Archiving and Communication System (PACS)-related features. Unlike many other proprietary EMR systems, Bahmni is open-source and allows feature customization, in order to meet the specific needs of resource-limited settings [12]. However, little is known about how physicians perceive and experience this EMR system, and the factors influencing their satisfaction or dissatisfaction within this resource-limited healthcare context.

Research conducted at SPHMMC and Ayder Hospitals in Ethiopia showed that healthcare providers have a generally positive perception of the SmartCare EMR system despite facing challenges during its implementation. However, it is important to note that the research did not specifically focus on physicians’ perspectives. Following this, SPHMMC switched from using the SmartCare EMR to the Bahmni EMR system. The research identified various concerns at SPHMMC, such as inadequate training, lack of follow-up, insufficient management commitment, problematic network infrastructure, and compatibility issues with hardware/software leading to system downtime and technical glitches. Considering these results, it is necessary to conduct additional research on enhancements in these areas to guarantee efficient utilization of EMR systems [13].

Bahmni’s integration of hospital management and clinical modules makes it ideal for resource-limited settings. Unlike SmartCare, which is tailored to Zambia’s national system, Bahmni’s flexible, community-driven model supports broader adaptability. Its offline functionality, lightweight deployment, and proven use in rural hospitals in India and Nepal further affirm its relevance in low-resource environments [13,14].

A 2015 study conducted at five government hospitals in Addis Ababa revealed low adoption of the SmartCare EMR among healthcare professionals, accompanied by widespread dissatisfaction with its service. Physicians, in particular, exhibited higher levels of discontent. Despite examining the SmartCare EMR system, the study did not successfully pinpoint the specific factors influencing physician perceptions and satisfaction with the system [15].

A more recent study carried out in 2021 at referral hospitals across Ethiopia’s Amhara region which explores physicians’ attitudes towards the Smart Care EMR systems found that over 58% of respondents scored above average on a Likert scale, similar to findings in North Gondar hospitals (54.6%), Aider referral hospital (56.1%), and Makkah, Saudi Arabia (52.8%). The study also identified predictors of good attitudes such as computer ownership, EMR training, literacy, prior experience, computer access at work, and involvement in EMR activities. However, the study fails to identify other factors such as system usability and clinical workflow integration, which can determine the perception of physicians towards EMR systems [14].

A 2019 literature review on the usability of EMR systems in Sub-Saharan Africa reported an overall usability score of 66% across five key metrics. The strongest contributor was ease of learning (71%), indicating that users could quickly adapt to the systems. Cognitive load (68%) and effectiveness (67%) also had a positive impact, while efficiency (64%) and user satisfaction (63%) were the weakest contributors. The review’s main limitation was its reliance solely on published literature, with no direct observation, system testing, or user interviews [16].

A qualitative study carried out in 2014 in Al Ain, UAE, found that physicians in primary healthcare centers were generally satisfied with the Cerner EMR system, despite initial implementation challenges. The most valued features were efficient management of laboratory and radiology orders/results, an error-reducing and time-saving electronic prescription system, clearer and higher-quality documentation, pre-filled notes that saved time, and an improved referral process that enhanced patient traceability and continuity of care. However, drawbacks included the system’s complexity, unsuitability for primary care, increased documentation burden, and longer patient waiting times. Other concerns were reduced eye contact with patients, potential confidentiality issues, difficulty quickly reviewing historical notes, and a sense of being monitored for performance. The study’s findings are limited to the Cerner EMR system and primary care settings and cannot be generalized to hospitals using the Bahmni EMR system [17].

A study carried out in 2009 on 358 physicians affiliated with a large, multi-specialty clinic located in the Midwest region of the USA indicated that physicians generally view EMR technology as highly important, particularly for functions like entry/display of diagnoses and medications, and prescription alerts. Despite the perceived importance, there is little evidence that physician characteristics such as age, and medical specialty significantly impact their perception of EMR functions [18].

In the present study, the aim was to investigate physicians’ perceptions and satisfaction with the EMR system at SPHMMC.

**Figure 1:**
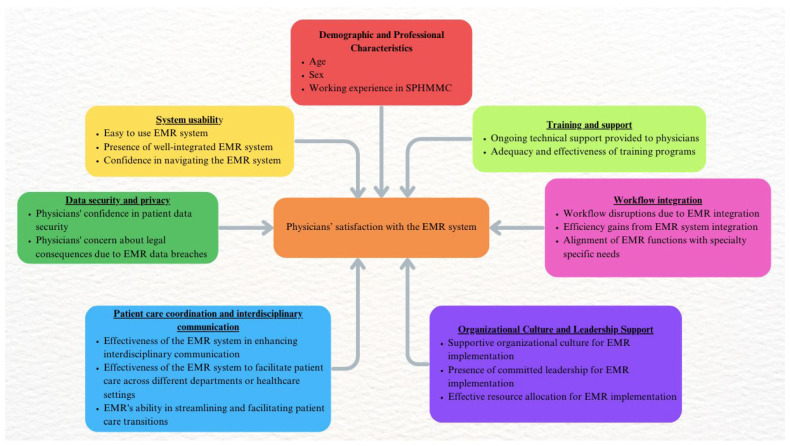
Conceptual framework for assessing physicians’ perception and satisfaction with the EMR system at SPHMMC as of 2024.

This conceptual framework served as a foundational guide for this study on physician perceptions and satisfaction with the EMR system in SPHMMC. It provided a structured approach to understanding and analyzing the complex relationships between various factors that influence physicians’ experiences with EMR systems. By mapping out these relationships, the conceptual framework helped in identifying key areas for investigation and formed the basis for hypothesis development and empirical testing. See details in Figure 1.

## 2. Methods

This present study took place at SPHMMC, one of the largest referral hospitals in the nation, where the Bahmni EMR system was implemented recently. The hospital offers specialized medical services to patients referred from across the country, educates medical and nursing students, and conducts both basic and applied research [13].

The study period encompassed 3 August 2024–31 January 2025, during which data collection, analysis, and interpretation were conducted to fulfill the objectives of the research. The quantitative data collection and qualitative interviews were conducted sequentially over the study period.

A facility-based mixed-method study design using a quantitative and qualitative approach was applied to capture all aspects of physician perception and satisfaction with the EMR system at SPHMMC. The quantitative component used a cross-sectional study design, where structured surveys were distributed electronically to a representative sample of physicians. The surveys collected numerical data on satisfaction levels, system usability, and demographic information. For the qualitative component, an exploratory research design was implemented, where in-depth interviews were conducted to explore physicians’ perceptions, challenges, and suggestions for improvement. This approach allowed for a deeper understanding of the factors influencing physician satisfaction that may not be captured through the quantitative data. The quantitative data provides a broad overview of satisfaction levels, while the in-depth interviews offer detailed insights into the physicians’ experiences and perceptions. This dual-method strategy provided a comprehensive understanding of the factors influencing physician satisfaction.

The study focused on physicians affiliated with St. Paul’s Hospital Millennium Medical College (SPHMMC), who actively use an Electronic Medical Record (EMR) system. It included a randomly selected group of clinical and academic physicians from various specialties and experience levels practicing in 2024. Eligible participants were medical doctors or interns at SPHMMC who regularly interacted with the EMR system in their clinical duties. Physicians affiliated with external institutions were excluded to maintain the study’s relevance to the hospital’s specific context.

- Operational definition

1. Physicians’ Perceptions

This refers to how physicians interpret and evaluate the Bahmni EMR system based on their experiences, expectations, and interactions with it. It encompasses their views on usability, relevance, efficiency, and impact on clinical workflows.

2. Perception

A broader construct that captures individual awareness, understanding, and interpretation of the EMR system’s features, benefits, and challenges. It is shaped by personal experience, institutional culture, and peer influence.

3. Satisfaction

Satisfaction reflects the degree to which physicians feel content with the EMR system’s performance, including ease of use, reliability, support, and its contribution to improving patient care and administrative efficiency.

4. Adoption of Bahmni EMR

Adoption refers to the extent and manner in which physicians integrate the Bahmni EMR into their daily clinical practice. It includes initial uptake, continued use, and the depth of engagement with its functionalities, influenced by training, system design, and institutional support.

- Quantitative research

The sample size was determined considering a level of confidence of 95% and margin of error (d) of 0.05. The estimated population proportion was assumed to be 50%, as no similar study has been conducted in our setting. Therefore, the total sample size for the quantitative study was 292.

In the absence of a complete centralized list of physicians, a stratified random sampling method was employed to ensure a representative sample across various departments and specialties at SPHMMC. Departments served as strata, and population sizes within each were determined through consultations with departmental heads or administrative staff. The total sample size was then proportionally allocated across these strata. Within each department, physicians were randomly selected to participate. Coordination with departmental heads facilitated the process, and invitations were delivered either in person or via the Telegram application.

Data was gathered through a structured questionnaire designed to assess physician perceptions and satisfaction with the EMR system at SPHMMC, covering topics such as usability, training, organizational support, data security, and the system’s impact on workflow and patient care. The questionnaire, based on validated tools and the literature, was pretested on a separate group of physicians to ensure clarity and accuracy before being distributed via Microsoft Forms. Descriptive statistics was employed to summarize demographic characteristics, such as age, gender, and years of experience, using frequencies, percentages, means, and standard deviations. Binary analysis explored relationships between independent and dependent variables using appropriate statistical tests. Multivariable logistic regressions analysis was utilized to identify factors that predict physician satisfaction with EMR systems.

- Qualitative research

For the qualitative aspect of this study, the sample size was eight physicians. This sample was deemed sufficient by achieving data saturation, where no new themes or insights were emerging from additional interviews. Criterion sampling, a purposive approach—was used to select physicians with direct and relevant experience using the EMR system, ensuring the collection of meaningful and insightful data. To capture diverse perspectives, stratified purposive sampling was also applied, incorporating participants from various specialties, experience levels, and roles within SPHMMC. Eligible participants were identified through their departments, and invitation letters outlining the study’s purpose, voluntary nature, and confidentiality measures were distributed via email and Telegram. Interested individuals contacted the research team to arrange interviews at times and locations convenient for them.

Semi-structured interviews were conducted using a refined interview guide to explore in-depth experiences with the EMR system. An interview guide was developed, based on an extensive literature review. It featured open-ended questions covering key topics such as overall perception and satisfaction, system usability, training and support, organizational culture and leadership support, data security and privacy, and the impact of the EMR system on workflow and patient care coordination. Interviews were conducted face-to-face, lasting approximately 30–45 min each. These interviews were audio-recorded with consent, transcribed, and translated, with all data anonymized for confidentiality. The analysis involved familiarization with the data, systematic coding, identifying and developing themes, patterns, and insights related to physician perceptions and experiences with EMR systems. The coding and analysis were executed using NVIVO software version 14:0.

For both aspects of this study, ethical protocols were strictly followed, including informed consent, assurance of participant rights, and robust data security measures to protect the integrity and privacy of all collected information.

## 3. Results

### 3.1. Presentation of Findings

#### 3.1.1. Demographics

The study involved 249 physicians, with an average age of 31.39 years (SD = 4.886), showing a young and diverse demographic as presented in Table 1.

The distribution of participants across departments in the quantitative survey reflects the general staffing pattern of physicians at SPHMMC. Internal Medicine, Obstetrics and Gynecology, and General Surgery were the most represented specialties, while specialties like urology and ophthalmology had fewer participants. Radiology, Obstetrics and Gynecology, and General Surgery reported the highest levels of previous EMR experience, while specialties like Anesthesiology and Ophthalmology had lower exposure, highlighting the need for targeted training in these areas. See details in Table 2.

Resident physicians primarily used it in combined settings, while specialists tended to focus on outpatient clinics. Notably, Emergency Medicine physicians used the system mainly in the adult emergency room, and Anesthesiology physicians accessed it in major ORs and inpatient wards.

#### 3.1.2. System Usability

The data analysis reveals that most physicians at SPHMMC have a positive inclination towards using the EMR system regularly, with 84.8% expressing interest in its daily use. However, a small proportion of physicians (7.6%) remain neutral or disagree with this intention. While the majority of physicians did not find the system overly complex, 20.1% expressed difficulty, with older physicians (40+) reporting more challenges in contrast to younger physicians (under 30).

Regarding the system’s ease of use, 61.0% of physicians agreed that it was user-friendly, though 10.4% disagreed, indicating some usability challenges. A mixed opinion emerged about the need for technical support, with 46.6% of physicians feeling confident using the system independently, while 25.7% felt they would require technical assistance. This need for support was more pronounced among older physicians.

The integration of the system’s functions was viewed positively by 54.2% of respondents, though 43.3% raised concerns about inconsistency. Furthermore, 64.3% of physicians believed the system was easy to learn, but 25.3% felt they needed to learn a lot before they could use it effectively.

**Figure 2:**
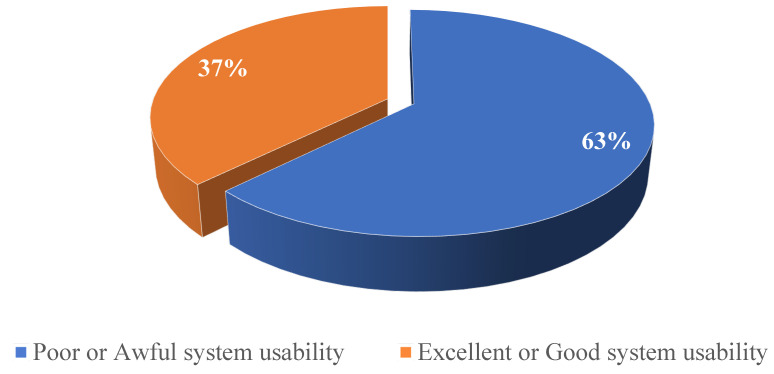
Interpretation of System Usability Scale (SUS) scores for the EMR system at SPHMMC—Visual representation of SUS scores assessing usability, ease of use, and user satisfaction with the EMR system, as of 2024.

In order to assess the usability of the EMR system at SPHMMC, the System Usability Scale (SUS) was used to quantify the perceived usability based on respondents’ feedback. The survey results indicate that 62.7% of respondents rated the system’s usability as “Poor or Awful” (95% CI: 56.2–68.7%), while 37.3% rated it as “Excellent or Good” (95% CI: 31.3–43.8%). See details in Figure 2.

#### 3.1.3. Training and Support

##### Training and Educational Opportunities for the EMR System

The evaluation of training and technical support for the EMR system at SPHMMC reveals significant gaps. Many respondents reported insufficient or no training, with 45.8% receiving no training and 41.8% feeling it was inadequate.

Satisfaction with EMR training modules varied among participants. Nearly half (49.6%, n = 67) were neutral, while 25.9% (n = 35) were dissatisfied to some degree. Conversely, 24.5% (n = 33) reported satisfaction, though this group was smaller than those who were neutral or dissatisfied. Only 9.6% considered the training adequate. Among those trained, 74.1% felt the training was insufficient, and 40% rated the training materials as ineffective. The training scheduling was also an issue, with only 25.2% finding it convenient.

**Figure 3:**
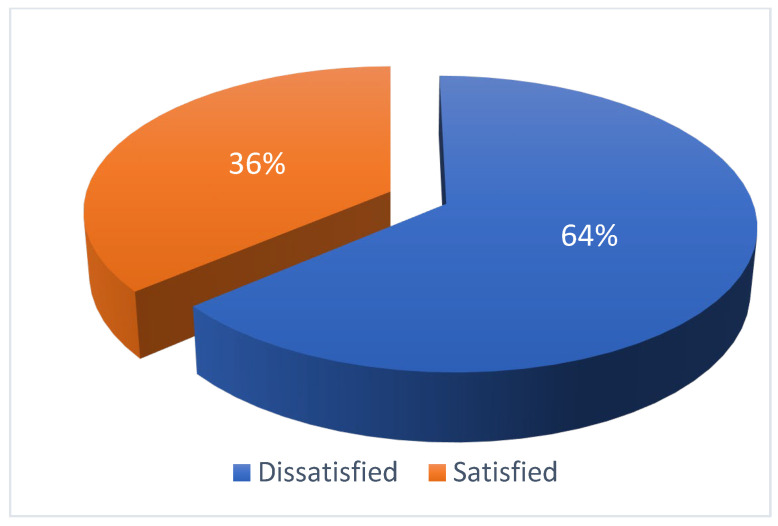
Overall satisfaction with EMR training and educational opportunities at SPHMMC—Visual representation of user satisfaction levels regarding the training and educational support provided for the EMR system, as of 2024.

The results of the survey show that 63.9% of respondents were dissatisfied (95% CI: 57.8–69.9%), while 36.1% expressed satisfaction (95% CI: 30.1–42.2%) with training and educational opportunities provided for EMR users at SPHMMC. See details in Figure 3.

##### Technical Support

Regarding technical support, 71.1% of respondents reported its availability, but 24.9% found it difficult to access. While 78% rated the responsiveness of support as adequate, 58.8% reported occasional difficulties in reaching support, and 10.4% faced frequent challenges.

**Figure 4:**
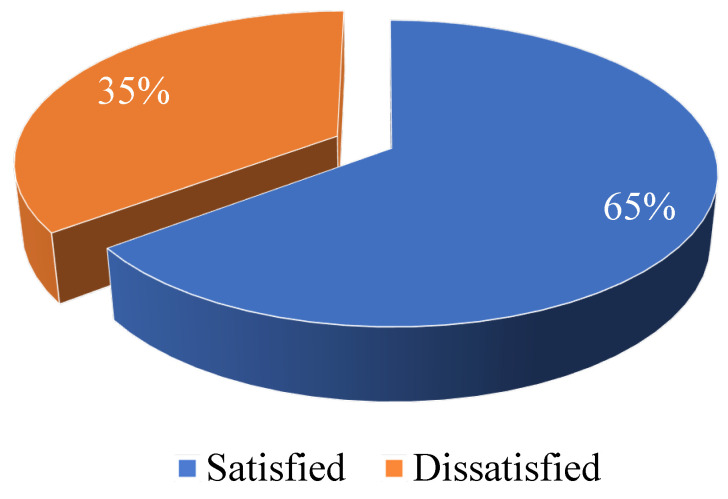
Overall satisfaction with technical support for EMR users at SPHMMC—Visual representation of physicians’ satisfaction levels with technical support for the EMR system, as of 2024.

Among 249 EMR users surveyed, 64.7% were satisfied with the technical support provided (95% CI: 58.2–70.3%), while 35.3% were dissatisfied (95% CI: 29.7–41.8%). See details in Figure 4.

#### 3.1.4. Organizational Culture and Leadership Support

**Figure 5:**
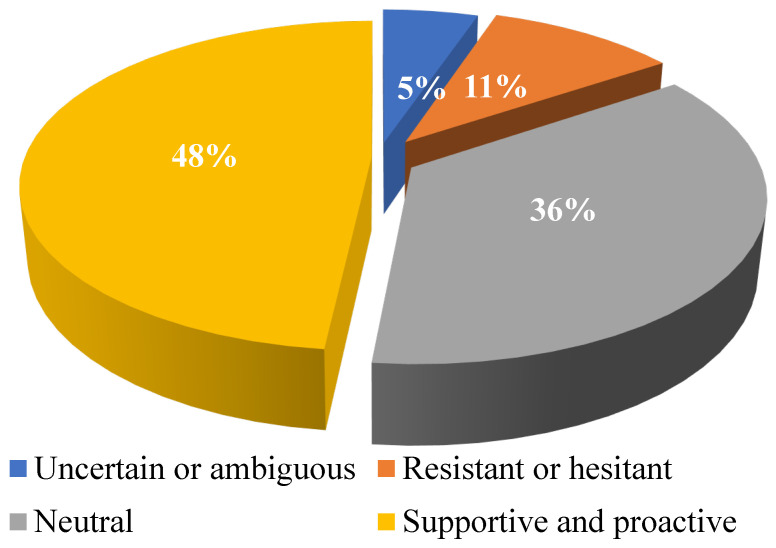
Physicians’ perceptions of SPHMMC’s organizational culture toward EMR adoption and integration—Visual representation of physicians’ views on the organizational culture supporting EMR adoption and integration, as of 2024.

The survey results on organizational culture and leadership support for EMR adoption at SPHMMC reveal areas that need attention. While 48.2% of respondents view the culture as supportive of EMR adoption, a significant portion (36.1%) remain neutral, and 10.4% perceive it as resistant. See details in Figure 5.

Communication regarding the importance of EMR was unclear for many staff, with 36.1% of participants reporting insufficient messaging. Additionally, 47.8% of respondents felt the culture of innovation was only moderately present.

**Figure 6:**
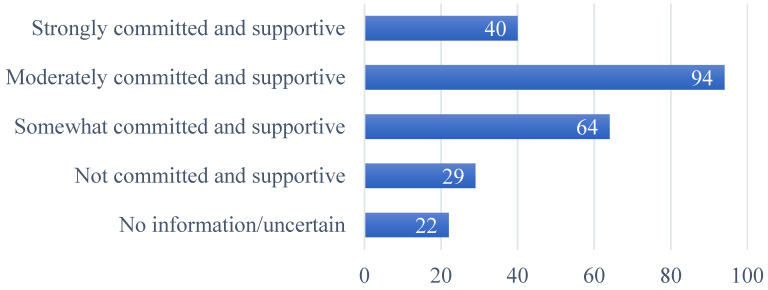
Physicians’ perceptions of leadership commitment and support for EMR implementation at SPHMMC—Visual representation of physicians’ views on leadership commitment and support for the EMR system implementation, as of 2024.

Perceptions of leadership’s commitment to EMR implementation at SPHMMC are mixed, with 79.6% acknowledging some level of support but varying in degree. Figure 6, below, summarizes these responses, highlighting areas for improvement in communication and engagement. 

Perceptions of policy clarity on EMR implementation vary, with nearly 47% acknowledging some clarity but 53% finding it lacking.

**Figure 7:**
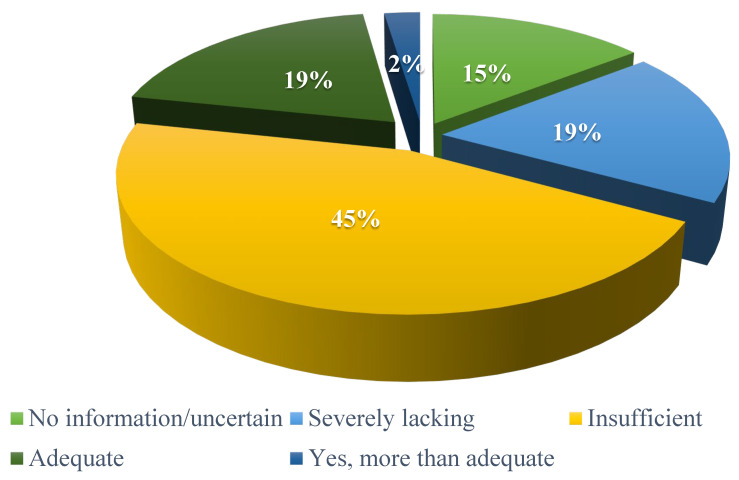
Physicians’ perceptions of resource allocation for EMR implementation at SPHMMC—Visual representation of physicians’ views on the adequacy of resources allocated for the EMR system, as of 2024.

Resource allocation was also a key issue, with 45.4% of respondents feeling resources were insufficient and 18.5% viewing them as severely lacking, suggesting a need for better resource distribution. Figure 7 outlines these responses. 

**Figure 8:**
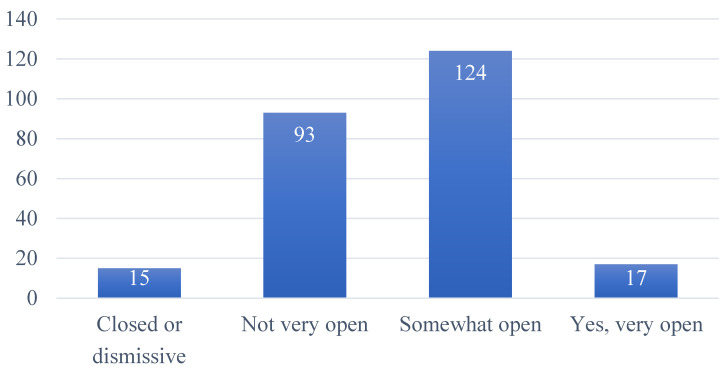
Physicians’ perceptions of an open and receptive environment for EMR feedback at SPHMMC—Visual representation of physicians’ views on the leadership openness and receptiveness to feedback regarding EMR system implementation, as of 2024.

Finally, feedback openness was mixed, with nearly 37.3% feeling that the environment was not open to feedback, underscoring the need for a more responsive approach to staff input during the EMR adoption process. See details in Figure 8.

#### 3.1.5. Workflow Integration

**Figure 9:**
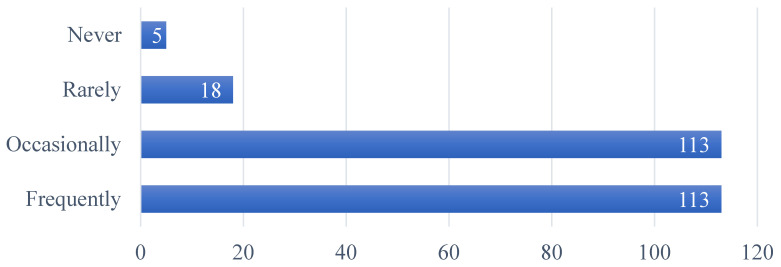
Frequency of disruptions in clinical workflow due to EMR system use at SPHMMC—Visual representation of the frequency of workflow disruptions caused by the EMR system, as of 2024.

Nearly 91% of physicians at SPHMMC report EMR-related disruptions, with frequent issues affecting Radiology, Internal Medicine, and ENT the most. Dermatology and Venereology report minimal impact. See details in Figure 9.

The main causes of disruptions include system freezes (78.7%) and integration issues (46.7%), followed by crashes (43.9%), time-consuming data entry (35.2%), and limited templates (25%). See details in Table 3.

**Figure 10:**
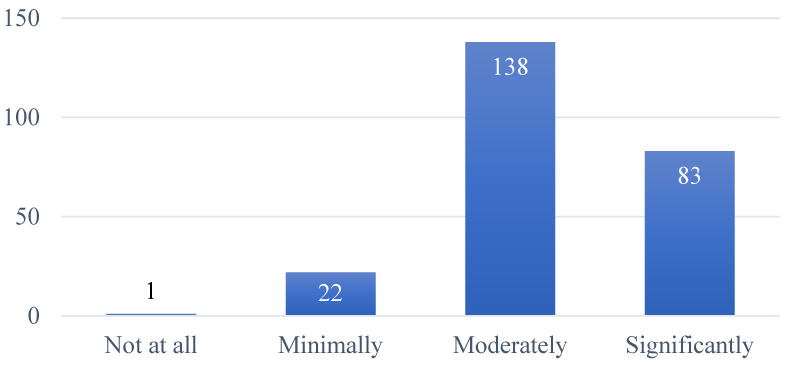
Extent of workflow disruptions affecting patient care at SPHMMC—Visual representation of the extent to which EMR-related workflow disruptions impact the ability to provide timely and effective patient care, as of 2024.

Over 90% of staff experience moderate to significant disruptions in patient care, with 34% reporting major challenges. See details in Figure 10.

Regarding efficiency, 65.8% note improvement, though 29.7% see no change, and 4.4% report declines. Workload perceptions vary: 50.6% report reduced workloads, 27.7% see no change, and 21.7% note increased burdens.

Specialty satisfaction levels vary, with 60% saying the system mostly meets their needs, while Internal Medicine and Emergency Medicine show the highest dissatisfaction.

**Figure 11:**
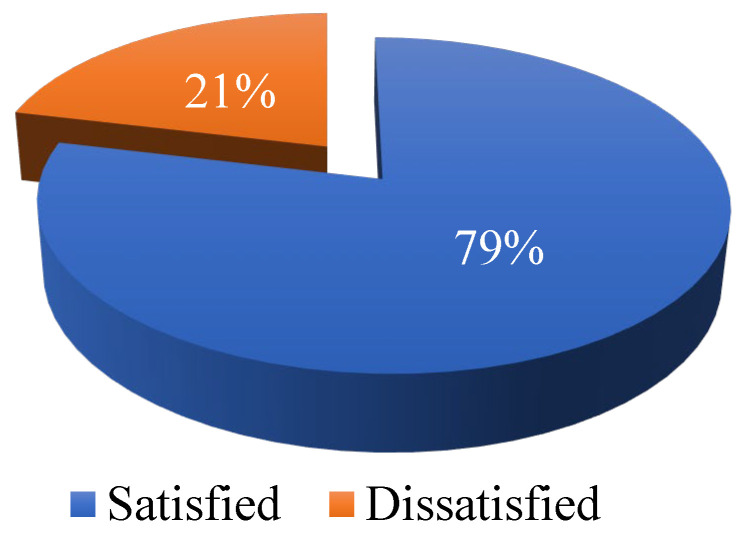
Physicians’ satisfaction with workflow integration of the EMR system at SPHMMC—Visual representation of percentage of physician satisfied with the integration of the EMR system into clinical workflows, as of 2024.

The survey results show that 78.7% expressed satisfaction with workflow integration (95% CI: 73.5–83.5%), while 21.3% reported dissatisfaction (95% CI: 16.5–26.5%). See details in Figure 11.

#### 3.1.6. Data Security and Privacy

Physicians at SPHMMC reported mixed confidence in EMR data security, with concerns about access control and legal risks due to perceived breaches. Table 4. below provides detailed insights into their responses. 

#### 3.1.7. Patient Care Coordination

Regarding patient care coordination, most physicians recognized the positive impact of the EMR system. Nearly half (47.4%) rated the system as moderately effective in facilitating care coordination, while 24.1% found it somewhat effective. In terms of care transitions, 53.4% felt the system provided some support, and 29.3% saw significant benefits.

**Figure 12:**
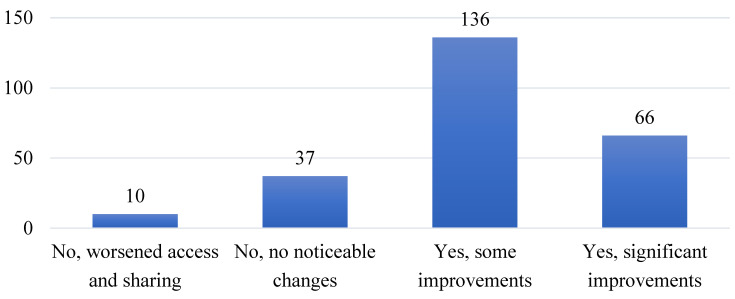
Improvements in accessing and sharing patient information across interdisciplinary care teams post-EMR implementation at SPHMMC—Visual representation of improvements in accessing and sharing patient information among interdisciplinary teams following EMR implementation, as of 2024.

Physicians also noted improvements in accessing and sharing patient information across interdisciplinary teams, with 54.6% observing some improvements and 26.5% reporting significant improvements. Despite these positive trends, a minority expressed dissatisfaction with the system’s effectiveness, suggesting areas for improvement. See details in Figure 12.

Responses on the effectiveness of EMR communication tools were mostly positive, with 86.8% rating them as somewhat to very effective. However, gaps in real-time communication support remain, as 54.6% reported only partial support.

**Figure 13:**
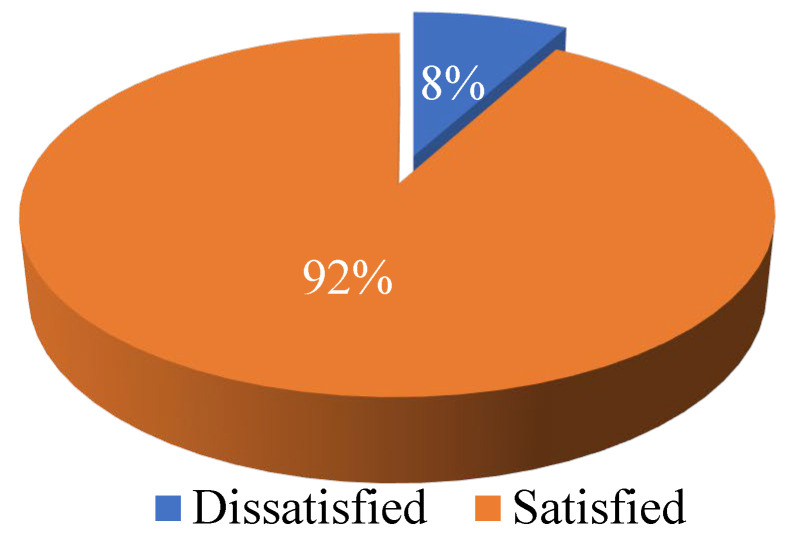
Physicians’ satisfaction with communication tools integrated into the EMR system at SPHMMC—Pie chart representing percentage of physicians satisfied with communication tools integrated into the EMR system, as of 2024.

Out of the 249 respondents, 92.0% reported satisfaction with communication tools integrated with the EMR system (95% CI: 88.8–95.6%), while 8.0% indicated dissatisfaction (95% CI: 4.4–11.2%). See details in Figure 13.

#### 3.1.8. Physicians’ Satisfaction with the EMR System at SPHMMC

Physician satisfaction with the EMR system was calculated by grouping survey responses into four key dimensions: training and support, system usability, workflow efficiency, and interdisciplinary communication. Each dimension score was derived as an average of related responses and combined into a composite overall satisfaction score, ensuring consistency with the study’s operational definition of physician satisfaction.

The training and support score reflected ratings across seven aspects, including the quality of training, effectiveness of materials, and accessibility of technical support. System usability was evaluated using SUS, with scores rescaled from 0 to 100, where higher values indicated better usability. Workflow efficiency was assessed based on four questions addressing the EMR system’s impact on disruptions, efficiency, workload, and specialty-specific needs. Interdisciplinary communication was measured through three questions evaluating the system’s ability to support communication and collaboration among care teams, focusing on information sharing, communication tools, and real-time support. The overall satisfaction index was then calculated as the average of these dimension scores, providing a comprehensive measure of physician satisfaction.

**Figure 14:**
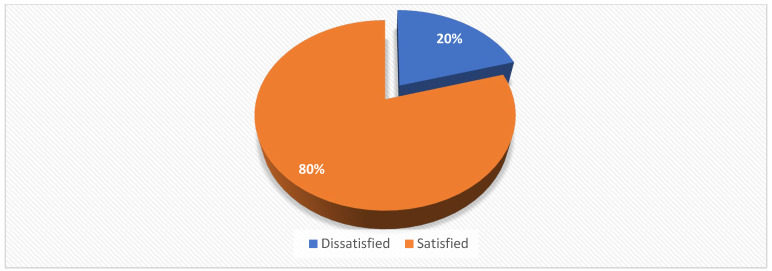
Overall physicians’ satisfaction with the EMR system at SPHMMC—Pie chart representing percentage of physicians that are satisfied with the overall aspects of EMR system, as of 2024.

The overall satisfaction data reveals a mean score of 17.38 and a median score of 17.78, indicating a slight skew towards higher satisfaction levels, with moderate variation (SD = 3.72) and a broad range of responses (6.90 to 27.33). Using 50% of the maximum possible score (14.09) as the cutoff for satisfaction, 79.5% (95% CI: 74.7–84.7%) of respondents were categorized as satisfied, while 20.5% (95% CI: 15.3–25.3%) were dissatisfied. This highlights a generally positive perception of the EMR system, though there remains a need for improvements to address areas of dissatisfaction. See details in Figure 14.

### 3.2. Data Analysis and Interpretation

#### 3.2.1. Binary Logistic Regression Analysis

Binary logistic regression analysis revealed several key factors influencing physician satisfaction with the EMR system at SPHMMC. See Table 5 below for details. 

#### 3.2.2. Multivariable Logistic Regression Analysis

Factors with *p*-values above 0.25 were included in a multivariable logistic regression model. The analysis highlighted several critical factors that significantly influence overall physician satisfaction with the EMR system at SPHMMC.

Physicians who found the EMR system easy to use were approximately seven times more likely to be satisfied (AOR: 7.298; 95% CI: 2.698–19.737, *p* < 0.001).

Physicians who found the EMR system well-integrated were nearly ten times more likely to be satisfied (AOR: 9.656; 95% CI: 2.878–32.396, *p* < 0.001).

Physicians who are confident in navigating the EMR system were nearly fifteen times more likely to be satisfied (AOR: 15.555; 95% CI: 5.668–42.687, *p* < 0.001).

Sufficient technical support was significantly associated with satisfaction, tripling the likelihood of positive feedback (AOR: 3.426; 95% CI: 1.24–9.468, *p* = 0.018).

Physicians who perceived the EMR system as effective in facilitating patient care across departments were nearly four times more likely to be satisfied (AOR: 3.927; 95% CI: 1.158–13.316, *p* = 0.028).

The details of these findings can be found in Table 6.

## 4. Qualitative Research Findings

The qualitative component included interviews with eight key informants, representing various roles and departments at SPHMMC. Participants comprised resident physicians, medical interns, and a specialist/consultant, with clinical experience ranging from less than eight months to five years. The departments represented included Emergency and Critical Care, Internal Medicine, Surgery, and medical interns, who have done rotations in various departments at the time of the interview. This diverse sample provided a broad perspective on the use of the Bahmni EMR system within the hospital. See Table 7 below for details 

A thematic analysis of feedback collected from these physicians regarding their experiences with the EMR system was organized into five themes:

1. Disconnect between the EMR system’s potential benefits and the reality of limited resources.
2. Challenges posed by the EMR system’s design and features on workflow efficiency.
3. Inadequate training and support hampering system mastery.
4. Data security and privacy concerns.
5. Patient care benefits and challenges.

### 4.1. Disconnect Between the EMR System’s Potential Benefits and the Reality of Limited Resources

This dominant theme, evident throughout the data, reveals a critical disconnect between the EMR system’s potential benefits and the reality of limited resources. The sources consistently cite inadequate infrastructure, including unreliable internet connectivity, insufficient computers, and unstable electrical power as major obstacles hindering the system’s effective use.

Many participants express frustration with frequent internet outages that disrupt workflow and force reliance on personal mobile devices and third-party applications like Telegram and Notion. One participant shared, “…*As I mentioned earlier, due to the network problem; we were unable to see the patient’s information while we were making rounds on patients. To overcome this problem, we were forced to use social media such as Telegram and other apps like Notion. Since these apps work on mobile internet; we were copying the full text or photo of the patient’s information from the EMR system to these applications and we were doing rounds. We also used to take photos of laboratory results from outside the hospital and upload them to the applications I mentioned*…” (KI 2-Medical Intern). This reliance raises concerns about incomplete patient information/data in clinical decision making and underscores the urgent need for reliable connectivity to ensure uninterrupted access to patient information.

The shortage of computers is another recurring concern, leading to delays, competition for access, and a sense of inadequacy in terms of resources. One intern emphasizes the imbalance: “*…The 4 computers we mentioned in the wards are to be used by 3 to 4 interns, 4 to 5 residents, 5 to 7 nurses and seniors…*” (KI 4-Medical Intern). This scarcity forces healthcare professionals to prioritize tasks, wait for their turn, or revert to paper-based methods, impacting efficiency and potentially compromising patient care.

The sources also point to inadequate leadership support, perceiving a lack of commitment to providing the necessary resources for the EMR system’s success. While leadership acknowledges the system’s importance, the perceived lack of concrete action to address resource gaps leaves many feeling disillusioned and unsupported. For example, one respondent remarked, “…*But the commitment of the management requires a lot of work. For example, I don’t think the management is committed to increasing the number of computers, setting up a fast network, and replacing the keyboards that are not working in some places*…” (KI 3-1st year resident physician).

### 4.2. Usability and Functionality Gaps Impacting Workflow

This theme highlights areas where the EMR system’s design and features create challenges for users, impacting workflow efficiency and user satisfaction. While the system’s ability to provide comprehensive patient information is acknowledged, participants express frustration with its cluttered interface, overwhelming amount of information, and missing functionalities.

Several sources criticize the lack of a clear and intuitive interface, making it difficult to quickly locate specific information or navigate through the system efficiently. One resident noted the difficulty in finding physician evaluations amidst extensive nursing notes, stating: “…*Nursing department will have to enter 20 pages worth of notes per newly admitted patients, and it will be very hard to find the next evaluation amidst all that information…*” (KI 6-2nd year resident physician).

The participants mentioned the absence of crucial functionalities, such as dedicated sections for consultations, MDT notes, and uploading external lab results, and this has forced users to rely on workarounds and compromises the system’s effectiveness. They also mentioned the lack of integration with the hospital laboratory system, which creates delays and frustration, as one intern explains: “…*When a doctor orders a lab investigation for a patient, the results should be sent to him/her when they come out but currently what we see is that the patients are being sent to bring the result…*” (KI 4-Medical Intern).

The limited mobile accessibility further restricts the system’s usability, particularly during rounds and in areas with poor internet connectivity. One specialist noted, “…*The system is not as accessible via mobile phones so that is one area of improvement*…” (KI 8-Internist). This limitation necessitates alternative solutions, such as using mobile data or transferring patient information to external apps, highlighting the need for improved mobile functionality to enhance access and workflow.

### 4.3. Inadequate Training and Support Hampering System Mastery

This theme highlights the crucial role of adequate training and ongoing support in facilitating a smooth transition and ensuring user proficiency with the EMR system. The sources consistently reveal a lack of comprehensive training, leaving many staff members feeling unprepared and reliant on self-learning, informal peer support, or trial and error.

One resident physician recounts their experience: “…*The first time I started working as a resident was in the OPD. There was only a 5-min talk about the EMR system, which was not very detailed, and a nurse there showed me how to use it. That is how I have been working with the EMR system so far*…” (KI 3-1st year resident physician). This lack of structured training leads to inconsistent understanding and usage of the system across departments, potentially increasing the risk of errors and hindering the system’s full potential.

While technical support is available, it is often reactive rather than proactive and not readily accessible outside regular working hours. One intern reported, “…*The IT team provides assistance to an extent through weekly visits. However, the team usually focuses on troubleshooting rather than enhancing system usage*…” (KI 7-Medical Intern). This limited support leaves staff members struggling to resolve technical issues independently, particularly during evenings and weekends. Another intern added, “…*But when we come to the support there are IT staff who come in early at 1 LT in the morning and leave at 12:30 to 1 LT in the evening and for any problems their phone numbers are written down in the wards. They come immediately when there are issues or try to solve it over the phone. Considering that, though not adequate, I would say the support is good*…” (KI 4-Medical Intern).

The sources emphasize the need for formal, comprehensive training programs that cater to different learning styles and user needs, as well as readily available technical support to address questions and resolve problems promptly.

### 4.4. Data Security and Privacy Concerns

This theme underscores the critical importance of safeguarding patient data within the increasingly interconnected digital healthcare landscape. The sources express significant concerns about the EMR system’s security measures, citing lack of logout enforcement and potential leaks due to the transfer of patient information to external applications.

The sources frequently mention the issue of doctors forgetting to log out of the EMR system, creating opportunities for unauthorized access to sensitive patient information. This recurring problem stems from a lack of user discipline and highlights a critical security vulnerability within the system. Several sources describe instances where healthcare providers, particularly doctors, neglect to log out after using shared computers. A resident expressed their concern on this matter, stating that “*…But what I have noticed is that many doctors log in and then forget to log out. Then, because there are so few computers, once that doctor’s account is opened, the EMR system can be accessed by other people. This allows another doctor to manipulate the information entered by one doctor, and orders can be written in the doctor’s name without his knowledge…*” (KI 3-1st year resident physician).

According to the key informants, the reliance on third-party applications like Telegram for communication and data sharing further amplifies security risks, as these platforms may not adhere to the same stringent privacy standards as healthcare-specific systems. One medical intern noted, “…*and sometimes when the internet breaks down during our rounds, we transfer the patient’s data to another unassociated application to make it accessible which may lead to data leak*…” (KI 4-Medical Intern). Thus, physicians have raised concerns about potential data leaks, and this underscores the need for secure, internal communication channels within the EMR system.

The sources advocate for enhanced security measures, including implementing automatic logout features as a crucial security measure to mitigate risks and ensure that patient data remains confidential and protected within the EMR system. They also stress the importance of raising awareness among healthcare professionals regarding data privacy best practices to foster a culture of responsible data handling and minimize potential breaches.

### 4.5. Patient Care Benefits and Challenges

This overarching theme reflects the complex and multifaceted impact of the EMR system on patient care at SPHMMC. While the sources acknowledge the potential benefits of the system, such as improved continuity of care, reduced wait times, and enhanced patient safety, they also identify significant implementation challenges that hinder the realization of these benefits.

Participants appreciate the centralized access to patient information and the elimination of physical files, which streamlines communication between healthcare providers and reduces the risk of lost or misplaced records. A consultant physician said, “…*There were some common issues that we would notice with regards to patients’ files and medical records being lost. Now that we have come to an electronic system it has decreased the chances of a patient’s data being lost.* …” (KI 8-Internist). The system also facilitates efficient order entry and prescription management, reducing errors and promoting patient safety.

However, the implementation challenges, including technical issues, usability gaps, and inadequate training, create potential negative impacts that must be addressed. Disruptions to rounds due to unreliable internet connectivity and the increased focus on documentation over direct patient interaction raise concerns about potential compromises in patient care. One physician commented, “…*Another is that it is not easy to access the patients’ vital signs, input and output levels, and their progress during the round, so the round has become partial. The complete patient information is not shared with the doctors*…” (KI 1-2nd year resident physician).

The transition to an electronic system also presents communication challenges, particularly for patients and attendants accustomed to paper-based methods. An intern recounts their experience, “…*we must note that patients and their attendants come from different educational backgrounds and levels of literacy. For example, when we inform them that we have sent the order* via *the system, how many of them truly understand what we mean? Patients are accustomed to the paper method, and they often become confused*…” (KI 7-Medical Intern). The sources stress the importance of patient education and engagement to ensure understanding and acceptance of the new system. Addressing these challenges and optimizing the system’s functionality are crucial to ensure that the EMR system ultimately fulfills its potential to enhance patient care.

## 5. Discussion

This research, exploring physicians’ perceptions and satisfaction with the newly implemented EMR system at SPHMMC, reveals a complex interplay of benefits and challenges. While physicians acknowledge the system’s potential in improving communication and patient care coordination, usability challenges, insufficient training and support, and workflow disruptions significantly hinder their overall experience and satisfaction.

The findings of this study at SPHMMC provide valuable insights into the physicians’ perception and satisfaction with the EMR system. These results align with and, in some cases, diverge from existing literature, offering a nuanced understanding of EMR adoption in resource-limited settings.

Physician satisfaction with EMR systems varies widely across settings, reflecting the influence of multiple factors. At SPHMMC, 79.5% of physicians were satisfied with the EMR system, highlighting its positive reception across dimensions like training and support, system usability, workflow efficiency, and interdisciplinary communication. This is notably higher compared to a study in Brazil, where 52.5% of primary care physicians reported satisfaction or partial satisfaction [19]. Conversely, studies in Ethiopia revealed more negative outcomes, with 61.4% of health professionals expressing dissatisfaction after three years of EMR implementation, citing persistent challenges [14]. In contrast, studies of fully functional EMR systems in other settings have reported high satisfaction rates, with 93% of physicians satisfied with comprehensive systems and 88% with basic ones [20]. These findings underscore the variability in EMR satisfaction across different settings, which can be attributed to a combination of healthcare system factors such as resource availability, healthcare delivery model (e.g., primary care vs. specialist care), stage of EMR implementation (early vs. mature), variations in training and support, and cultural and contextual factors.

Studies on EMR systems reveal that training quality plays a crucial role in shaping physician satisfaction and system acceptance. In U.S. Family Practice Residency Programs, while residents acknowledged EMR benefits, frustration and ambivalence arose due to training inadequacies, emphasizing the importance of relevance over duration [21]. Similarly, in Addis Ababa’s five government hospitals, low EMR utilization and dissatisfaction were linked to insufficient training, limited technical support, and system challenges [14]. Conversely, Stanford Children’s Health demonstrated the transformative impact of a well-structured, comprehensive training program, leading to high physician satisfaction through tailored content and conducive learning environments [22]. Aligning with these findings, research at SPHMMC revealed that a significant 45.8% of physicians reported receiving no training, and 41.8% felt the training was inadequate, on the Bahmni EMR system, which led to unpreparedness and reliance on informal learning, although satisfaction with training opportunities did not significantly affect overall EMR satisfaction. Together, these findings highlight that effective, context-specific training is critical to optimizing EMR adoption and satisfaction.

Studies across various contexts underscore the critical role of technical support in the successful implementation and acceptance of EMR systems. In a U.S. pediatric emergency department and associated clinics, satisfaction with ambulatory EMR systems was strongly linked to the availability of robust technical support [23]. Similarly, in Ethiopia’s St. Paul’s and Ayder hospitals, key barriers to EMR implementation included inadequate training, poor ICT infrastructure, and insufficient technical support, with 29.2% and 38% of respondents, respectively, highlighting these as major challenges [13]. Supporting these findings, this research at SPHMMC revealed that sufficient technical support significantly enhanced satisfaction with the Bahmni EMR system, tripling the likelihood being satisfied with the EMR system. These findings collectively highlight that effective technical support and training are pivotal for overcoming barriers, improving user satisfaction, and ensuring the success of EMR systems.

Research findings consistently highlight the pivotal role of system usability and integration in shaping physician satisfaction with EMR systems. At Michigan State University, providers reported high satisfaction with usability despite mixed overall ratings, though initial enthusiasm declined over time, emphasizing the need for continuous improvements [24]. Similarly, findings from SPHMMC reveal that perceived ease of use significantly increased satisfaction odds by 7.3 times, while a well-integrated system raised odds nearly tenfold, and confidence in system navigation boosted satisfaction over fifteen-fold. But this study may not reflect long-term trends, and future research is required to study how these factors affect physicians’ satisfaction with EMR system over longer period of time.

At SPHMMC, usability challenges persist, as 62.7% of respondents rated usability as “Poor or Awful,” with older physicians disproportionately perceiving the system as overly complex. This can be explained by limited technological experience, resistance to changing established habits, and inadequate training. Addressing these challenges requires targeted training and user-friendly system design. Only 54.2% of physicians viewed the system’s integration positively, with 43.3% expressing concerns about inconsistencies that could confuse users and lengthen the learning curve. These findings demonstrate that while usability and integration significantly enhance satisfaction, addressing complexity and functional inconsistencies is essential for widespread adoption and sustained positive experiences.

Organizational support plays a critical role in the adoption and satisfaction with EMR systems, as evidenced by research in Iran, where management support significantly influenced physicians’ perceptions of EMR usefulness and ease of use [25]. Similarly, findings at SPHMMC underscore the need for improved organizational culture to enhance EMR adoption. While many physicians view the culture as supportive, a notable portion remains neutral or resistant, often due to unclear communication about EMR importance and moderate encouragement of innovation. Perceptions of leadership commitment are inconsistent, with insufficient visibility and resource allocation being major concerns. Physicians frequently reported a disconnect between EMR functionality and available resources, such as computers and technical support, leading to frustration and disillusionment. Additionally, mixed openness to feedback highlights the need for leadership to adopt a more responsive and inclusive approach. These findings demonstrate that fostering a supportive organizational culture, through clear communication about benefits of EMR through regular meetings and training, resource investment, active leadership engagement by having leaders actively engage with staff, and promoting a culture of innovation by incentivizing staff suggestions, is essential to improving physician satisfaction and successful EMR implementation.

The integration of EMR systems into clinical workflows profoundly influences physician satisfaction and productivity, with disruptions and system limitations posing significant challenges. At SPHMMC, 91% of physicians reported EMR-related disruptions, with radiology, internal medicine, and ENT specialties most affected. These disruptions stemmed from issues such as system freezes, integration failures with other healthcare platforms, crashes, time-intensive data entry, and limited templates meeting the need of each specialty. While 50.6% of physicians observed reduced workloads post-implementation, 21.7% reported increased burdens, reflecting mixed perceptions of EMR impact on workflow. Similarly, dissatisfaction was highest among internal medicine and emergency medicine physicians regarding the system’s ability to meet specialty-specific needs. These findings align with broader research showing that while EMRs can enhance productivity, they may also introduce complexities that increase workload [26]. In a study conducted on primary care physicians, additional concerns, such as the potential intrusion on the doctor–patient relationship, data entry pressures, and the inflexibility of EMR systems, further illustrate how workflow integration challenges can hinder satisfaction and efficiency [8]. Addressing these barriers by improving system customization, reducing disruptions, and aligning functionality with clinical demands is crucial for optimizing EMR adoption and user experience.

Effective communication and care coordination are pivotal factors influencing physician satisfaction with EMR systems, as highlighted by the SPHMMC research findings. Physicians who perceived the system as enhancing communication workflows and streamlining care transitions reported higher satisfaction levels, with 92% expressing contentment with the integrated communication tools. These tools fostered improved interdisciplinary collaboration and information exchange, significantly contributing to positive perceptions of the system. Additionally, 54.6% of physicians noted moderate improvements in accessing and sharing patient information across teams, while 26.5% observed significant enhancements in care coordination. These benefits align with findings from a study in Manitoba, where an EMR system reduced communication issues and improved prescribing safety between pharmacists and prescribers [27]. Together, these insights emphasize the importance of EMR systems in facilitating seamless communication and collaboration, ultimately driving physician satisfaction and supporting more effective and coordinated patient care.

Similarly to the present study, another qualitative study carried out in Al Ain acknowledged that EMRs offered patient care benefits, such as improved continuity of care and reduced lost charts; the Al Ain study specifically lauded the system’s management of laboratory and radiology orders and results as its “strongest point” and praised electronic prescriptions for reducing errors and saving time. Conversely, both studies extensively detailed significant challenges: physicians in Al Ain and SPHMMC found the systems time-consuming, impacting their practice and patient communication, often leading to increased waiting times. Usability and complexity issues were prominent, with SPHMMC physicians reporting a cluttered interface, overwhelming information, and missing functionalities, while Al Ain physicians noted the system was not designed for primary care and had difficulties with proper coding for diagnoses. Inadequate training and support were common frustrations; SPHMMC physicians reported insufficient formal training, relying on self-learning and peer support, with technical support often reactive, while Al Ain physicians desired individualized and updated training. Concerns over data security and privacy were also shared, stemming from issues like doctors forgetting to log out of shared computers in SPHMMC and the general sentiment of “no confidentiality” in Al Ain where “anybody can open the file”. Uniquely, SPHMMC highlighted a critical disconnect between EMR potential and limited resources, citing unreliable internet, insufficient computers, and unstable electricity, alongside a perceived lack of leadership commitment to these resource gaps. In contrast, the Al Ain study uniquely found that physicians perceived the EMR as a “significant threat” due to its use for auditing their documentation and performance [17].

Overall, physician perceptions of the EMR system at SPHMMC are mixed. They acknowledge the potential of the system to improve patient care coordination and communication, but significant usability challenges, inadequate training and support, and workflow disruptions hinder their overall experience. Addressing these challenges is crucial to improving perceptions and fostering greater acceptance of EMR systems.

### 5.1. Strengths of the Study

The mixed-methods design enabled a rich exploration of Bahmni EMR implementation at SPHMMC, blending quantitative trends with qualitative physician insights. This context-specific approach allowed for a nuanced understanding of system use and organizational culture, enhancing the relevance of findings to the institution. The integration of diverse data sources strengthened the study’s credibility and depth of analysis.

### 5.2. Limitations of the Study

The study’s focus on SPHMMC physicians limits generalizability to other settings with different EMR systems or cultures. Reliance on self-reported data and volunteer interviews introduces potential bias. The short data collection period and cross-sectional design restrict insights into long-term trends and causal relationships, while the incomplete EMR rollout further constrains broader applicability of the findings.

## 6. Conclusions

This study revealed mixed physician perceptions of the EMR system at SPHMMC, with concerns around usability, training, infrastructure, and leadership communication. Despite 91% reporting workflow disruptions, most acknowledged benefits like improved care continuity, reduced chart loss, and enhanced patient safety. High satisfaction with technical support and workflow integration suggests strong potential. To fully realize EMR advantages, proactive steps are needed: redesigning the interface, expanding training, strengthening infrastructure, and improving leadership engagement. Addressing these gaps will enhance system adoption, reduce resistance, and optimize clinical workflows, ultimately improving healthcare delivery and patient outcomes across departments.

## Figures and Tables

**Table 1:** Demographic and professional characteristics of physicians at SPHMMC.

		Frequency	Percent
Age group	Young physicians (<30)	108	43.4%
	Middle-aged physicians (30–39)	119	47.8%
	Old-aged physicians (40+)	22	8.8%
Sex	Male	166	66.7%
	Female	83	33.3%
Professional role	Intern	25	10.0%
	General practitioner	3	1.2%
	Year I resident	61	24.5%
	Year II resident	58	23.3%
	Year III resident	32	12.9%
	Year IV resident	15	6.0%
	Specialist/Consultant	22	8.8%
	Fellow	8	3.2%
	Sub-specialist	25	10.0%
Working experience in SPHMMC	<1 yr	82	32.9%
	>10 yrs	10	4.0%
	1–5 yrs	127	51.0%
	6–10 yrs	30	12.0%
Previous experience with EMR outside SPHMMC	Yes	153	61.4%
	No	96	38.6%

**Table 2:** Work settings for accessing the EMR system at SPHMMC—Frequency and percentage of physicians accessing the EMR system across clinical settings (outpatient clinics, inpatient wards, ORs, and emergency rooms) as of 2024.

	Frequency	Percent
Both at outpatient clinics and inpatient wards	68	27.3%
Mostly at outpatient clinics	56	22.5%
Only at outpatient clinics	39	15.7%
At major ORs, outpatient clinics, and inpatient wards	23	9.2%
At adult emergency room, major ORs, outpatient clinics, and inpatient wards	19	7.6%
At adult emergency room only	14	5.6%
At adult emergency room, outpatient clinics, and inpatient wards	14	5.6%
At major ORs and mostly at outpatient clinics	11	4.4%
At adult emergency room and mostly at outpatient clinics;	3	1.2%
Only at outpatient clinics and major ORs	2	0.8%

**Table 3:** Challenges and workflow disruptions due to EMR system use at SPHMMC. Frequency and percentage distribution of responses on challenges, including delayed response times, system freezes, integration difficulties, and other disruptions, as of 2024.

Response	Selected	
	Frequency	Percent
There is delayed response times or system freezes	192	78.7%
There are difficulties in integrating EMR systems with other healthcare platforms (e.g., laboratory information system, radiology information system) or exchanging information with external providers (e.g., refer outs and ins)	114	46.7%
There are unexpected system crashes, maintenance periods, or technical glitches.	107	43.9%
There is a need for extensive data entry, including documentation of patient encounters, prescriptions, and orders, which can be time-consuming	86	35.2%
There are limited documentation templates which doesn’t have customization and flexibility to accommodate needs of different specialties or individual patient cases	61	25.0%
There are excessive amounts of clinical data, such as redundant or irrelevant information.	37	15.2%
There are overwhelming numbers of pop-up alerts or notifications, such as medication alerts or clinical decision support prompts.	15	6.1%
WiFi connection and network issues	4	1.6%
Power outages disrupting EMR system functionality	4	1.6%
Limited computer availability hindering efficient EMR usage	3	1.2%
Nighttime system downtime in the laboratory and pharmacy necessitates paper-based investigations and prescriptions	3	1.2%
Restriction on data editing after an hour leads to challenges in correcting errors or updating records	2	0.8%
Challenges in accessing patient data during rounds and emergencies hinder timely decision-making.	2	0.8%
Unavailability of endoscopy results	1	0.4%
Multiple open windows for different functions create confusion and risk duplication of notes	1	0.4%

**Table 4:** Physicians’ confidence and concerns regarding EMR data security at SPHMMC—Frequency and percentage distribution of responses on confidence in EMR security measures, perceived unauthorized access, and concerns about legal consequences, as of 2024.

	Frequency	Percent
**Confidence in EMR security measures**		
Not confident at all	11	4.4%
Not very confident	29	11.6%
Neutral	96	38.6%
Confident	82	32.9%
Very confident	31	12.4%
**Perceived instances of unauthorized access**		
No, never	172	69.1%
Yes, occasionally	58	23.3%
Yes, frequently	19	7.6%
**Concerns about legal consequences resulting from breaches of patient data confidentiality or privacy within the EMR system**		
Not concerned at all	8	3.2%
Not very concerned	25	10.0%
Neutral	73	29.3%
Concerned	93	37.3%
Very concerned	50	20.1%

**Table 5:** Factors associated with overall physicians satisfaction with the EMR system at SPHMMC—Binary logistic regression analysis of variables influencing overall satisfaction as of 2024.

Variables	Responses	Overall Satisfaction with the EMR System		*p* Value	COR	95% CI for COR	
		Dissatisfied	Satisfied			Lower	Upper
Age group	Young physicians (<30)	16 (6.4%)	92 (36.9%)	0.022	3.286	1.187	9.094
	Middle-aged physicians (30–39)	27 (10.8%)	92 (36.9%)	0.178	1.947	0.739	5.13
	Old-aged physicians (40+)	8 (3.2%)	14 (5.6%)	1.000	1		
Working experience in SPHMMC	Less than 1 year	18 (7.2%)	64 (25.7%)	0.065	3.556	0.926	13.652
	1–5 years	20 (8%)	107 (43%)	0.013	5.35	1.417	20.196
	6–10 years	8 (3.2%)	22 (8.8%)	0.180	2.75	0.626	12.085
	More than 10 years	5 (2%)	5 (2%)	1.000	1		
Sex	Male	40 (16.1%)	126 (50.6%)	1.000	1		
	Female	11 (4.4%)	72 (28.9%)	0.049	2.078	1.004	4.301
Easy to use EMR system	Disagree	31 (12.4%)	27 (10.8%)	1.000	1		
	Agree	20 (8%)	171 (68.7%)	<0.001	9.817	4.91	19.637
Presence of well-integrated EMR system	Disagree	47 (18.9%)	67 (26.9%)	1.000	1		
	Agree	4 (1.6%)	131 (52.6%)	<0.001	22.974	7.94	66.472
Confidence in navigating the EMR system	Disagree	40 (16.1%)	29 (11.6%)	1.000	1		
	Agree	11 (4.4%)	169 (67.9%)	<0.001	21.191	9.76	45.992
Supportive organizational culture for EMR implementation	Bad	39 (15.7%)	90 (36.1%)	1.000	1		
	Supportive and Proactive	12 (4.8%)	108 (43.4%)	<0.001	3.9	1.93	7.892
Presence of committed leadership for EMR implementation	Not committed and supportive	19 (7.6%)	32 (12.9%)	1.000	1		
	Committed and supportive	32 (12.9%)	166 (66.7%)	0.001	3.08	1.56	6.093
Effective resource allocation for EMR implementation	Insufficient or severely lacking	42 (16.9%)	153 (61.4%)	1.000	1		
	Adequate or more than adequate	9 (3.6%)	45 (18.1%)	0.434	1.373	0.62	3.033
Overall satisfaction with training and educational opportunities provided to EMR users	Dissatisfied	38 (15.3%)	121 (48.6%)	1.000	1		
	Satisfied	13 (5.2%)	77 (30.9%)	0.079	1.86	0.93	3.714
Overall satisfaction with technical support provided to EMR users	Dissatisfied	29 (11.6%)	59 (23.7%)	1.000	1		
	Satisfied	22 (8.8%)	139 (55.8%)	<0.001	3.106	1.65	5.845
Physicians’ confidence in patient data security	Not confident	32 (12.9%)	104 (41.8%)	1.000	1		
	Confident	19 (7.6%)	94 (37.8%)	0.193	1.522	0.81	2.865
Physicians’ concern about legal consequences due to EMR data breaches	Not concerned	26 (10.4%)	80 (32.1%)	1.000	1		
	Concerned	25 (10%)	118 (47.4%)	0.175	1.534	0.83	2.846
Workflow disruptions due to EMR integration	Frequent or occasional disruptions	50 (20.1%)	176 (70.7%)	1.000	1		
	Rare or no disruptions	1 (0.4%)	22 (8.8%)	0.077	6.25	0.82	47.516
Efficiency gains from EMR system integration	No improvement in efficiency	27 (10.8%)	58 (23.3%)	1.000	1		
	Improved efficiency	24 (9.6%)	140 (56.2%)	0.002	2.716	1.45	5.095
Alignment of EMR functions with specialty specific needs	Not aligned	32 (12.9%)	68 (27.3%)	1.000	1		
	Aligned	19 (7.6%)	130 (52.2%)	<0.001	3.22	1.70	6.1
Effectiveness of the EMR system in enhancing interdisciplinary communication	Not effective	13 (5.2%)	20 (8%)	1.000	1		
	Effective	38 (15.3%)	178 (71.5%)	0.005	3.045	1.39	6.65
Effectiveness of the EMR system to facilitate patient care across different departments or healthcare settings	Not effective	13 (5.2%)	15 (6%)	1.000	1		
	Effective	38 (15.3%)	183 (73.5%)	0.001	4.174	1.84	9.484
EMR’s ability in streamlining and facilitating patient care transitions	No	19 (7.6%)	24 (9.6%)	1.000	1		
	Yes	32 (12.9%)	174 (69.9%)	<0.001	4.305	2.12	8.757

**Table 6:** Multivariable logistic regression analysis of factors influencing physicians’ satisfaction with the EMR system at SPHMMC, 2024.

Variables	Responses	Overall Satisfaction with the EMR System		COR	*p* Value	AOR	95% CI for AOR	
		Dissatisfied	Satisfied				Lower	Upper
Easy to use EMR system	Disagree	31 (12.4%)	27 (10.8%)	1	1	1		
	Agree	20 (8%)	171 (68.7%)	9.817	<0.001	7.298	2.698	19.737
Presence of well-integrated EMR system	Disagree	47 (18.9%)	67 (26.9%)	1	1	1		
	Agree	4 (1.6%)	131 (52.6%)	22.974	<0.001	9.656	2.878	32.396
Confidence in navigating the EMR system	Disagree	40 (16.1%)	29 (11.6%)	1	1	1		
	Agree	11 (4.4%)	169 (67.9%)	21.191	<0.001	15.555	5.668	42.687
Overall satisfaction with technical support provided to EMR users	Dissatisfied	29 (11.6%)	59 (23.7%)	1	1	1		
	Satisfied	22 (8.8%)	139 (55.8%)	3.106	0.018	3.426	1.24	9.468
Effectiveness of the EMR system to facilitate patient care across different departments or healthcare settings	Not effective	13 (5.2%)	15 (6%)	1	1	1		
	Effective	38 (15.3%)	183 (73.5%)	4.174	0.028	3.927	1.158	13.316

**Table 7:** Roles, departments, and experience levels of key informants involved in the study, as of 2024.

Key Informant	Role	Department	Experience at SPHMMC
Informant 1	2nd year resident physician	Emergency and Critical Care	3 years and 4 months
Informant 2	Medical intern	Unspecified	1 year and 3 months
Informant 3	1st year resident physician	Internal Medicine	Less than 8 months
Informant 4	Medical intern	Unspecified	1 year and 3 months
Informant 5	Medical intern	Unspecified	1 year and 3 months
Informant 6	2nd year surgical resident	Surgery	5 years
Informant 7	Medical intern	Unspecified	1 year and 3 months
Informant 8	Specialist/Consultant	Internal medicine	5 years

## Data Availability

The original contributions presented in this study are included in the article/Supplementary Material. Further inquiries can be directed to the corresponding authors.

## Supplementary Materials

The following supporting information can be downloaded at: https://www.mdpi.com/article/10.3390/healthcare14152379/s1. Research data.

## Abbreviations

EMR	Electronic Medical Record
ENT	Ear, Nose, and Throat (medical specialty)
MDT	Multidisciplinary Team
OR	Operating Room
PACS	Picture Archiving and Communication System
SPHMMC	St. Paul’s Hospital Millennium Medical College
SUS	System Usability Scale
USA	United States of America
